# Contemporary Theory and Research

**Published:** 1893-07-08

**Authors:** 


### Contemporary Theory and Research.

### STERESOL.

Dr. Berlier, Professor of the School of Medicine at Grenoble, communicated at the last meeting of the Académie de Médicine very interesting particulars of a new remedy for use in affections of the mucous membrane and of the skin. It consists of an antiseptic varnish, steresol, able to adhere closely to the mucous membrane and skin. By varied experiments Dr. Berlier proves the powerfully bactercide action of steresol and shows that the phenol, which is the active constituent in it, only evaporates completely from the coat of varnish at the end of twenty-four hours. Applied on the throat steresol remains in place several hours, and resists the movements of deglutition. Its application is not painful, and is followed by no unpleasant effects. Employed in cases of diphtheria in the Trousseau Hospital during three months the proportion of cures was 81 per cent. Steresol modifies injured surfaces and preserves adjoining portions of the skin from contagion.

### FEMALE SANITARY INSPECTORS.

Dr. Wynter Blyth, Medical Officer of Health for the Parish of Marylebone, in making a plea for more sanitary inspectors in his monthly report, urgently recommends that two at least shall be females. He writes: "The duties of such female officers would be mainly, though not entirely, to carry out the sections of the Factory and Workshops Act relating to the employment of women; and investigation shows that a very large number of women are, as a fact, employed both as out and in workers in the dressmaking, millinery, and other occupations. Another legitimate use of such officers would be the superintendence of what may be called personal disinfection; to illustrate the meaning of this an example may be given. A tailor working at home was found to be suffering from small-pox, and promptly removed to the hospital. His wife in the same room possessed only the clothes she had on her back; obviously what was required here was a female inspector, who could have taken her to the shelter, stripped her, wrapped her up in a blanket, and then have had her clothes disinfected. All articles in the room have been dealt with, but this poor woman and her clothes, possibly, nay probably, infectious, for want of female assistance and appliances, have been left alone. Such an instance as the above must not be considered isolated or unusual; thousands of women in the parish only possess the clothes they usually wear, and it is one of the defects of our system of disinfection that these clothes, when contaminated from actual nursing of infectious sick children, can only occasionally be dealt with. The Public Health Conveniences Sub-Committee are also unanimously of opinion that the public conveniences for ladies should be under the supervision of a paid female officer; in short, there are numerous perfectly legitimate functions in connection with sanitary work that a woman alone can do efficiently, and I am convinced that when the idea becomes more familiar local authorities in the metropolis generally will give it practical effect. It is objected that the proper kind of woman is not likely to be found; this actual experience can alone show."

### FACTORY INSPECTION.

The Report of the Chief Inspector of Factories and Workshops details the revised rules in force in white lead works and the difficulties experienced in enforcing them. Paint and colour works and iron plate enamelling works have now been placed under similar regulations with the design of lessening the injurious effects to the workmen of these occupations. The special rules for lucifer match factories now require every case of necrosis to be reported to the factory inspector, and amongst other precautions forbid the employment of any person in the processes of mixing, dipping, or drying, after the extraction of a tooth, without a medical certificate that the jaw is healed; while all persons complaining of toothache or swelling of the jaw are to be examined by a medical man at the expense of the employer.

### A PROBLEM IN VACCINATION.

Dr. Calmette, Director of the Institute of Bacteriology and Vaccine of Saïgou in the district of Cochin China, has recently solved a problem which has for some time occasioned considerable difficulty in the operations of the institute. It was found that the vaccine of the heifer tended to become continually weaker and less efficacious in its passage from one animal to another, all more or less in a suffering state on account of their importation into those latitudes. It occurred to Dr. Calmette that by the substitution of the buffalo of the country for the heifer this deterioration might be avoided, and the result greatly surpassed his expectations. Fresh vaccine matter was sent from Paris with which the buffaloes were inoculated, and the strength of the lymph thus obtained was so well maintained that from 205 animals 8,676 tubes of vaccine matter were procured, sufficient not merely for the immediate district, but also for the districts of Madras, Manilla, and Singapore.

